# Vitamin D serum levels and non-melanoma skin cancer risk

**DOI:** 10.7717/peerj.12234

**Published:** 2021-09-24

**Authors:** Carolina Morgado-Águila, Guadalupe Gil-Fernández, Orlando Rafael Dávila-Villalobos, Jesús Pérez-Rey, Purificación Rey-Sánchez, Francisco José Rodríguez-Velasco

**Affiliations:** 1 Department of Plastic and Reconstructive Surgery, Cáceres University Hospital Complex, Extremadura Health Service, Cáceres, Extremadura, Spain; 2 Department of Nursing, Faculty of Medicine and Health Sciences, University of Extremadura, Badajoz, Extremadura, Spain; 3 Department of Gynecology and Obstetrics, Cáceres University Hospital Complex, Extremadura Health Service, Cáceres, Extremadura, Spain; 4 Department of Public Health, Extremadura Health Service, Extremadura Health Service, Cáceres, Extremadura, Spain; 5 Department of Nursing, Faculty of Nursing and Occupational Therapy, University of Extremadura, Cáceres, Extremadura, Spain

**Keywords:** Basal cell carcinoma, Skin cancers, Squamous cell neoplasms, Vitamin D3

## Abstract

**Background:**

Skin cancer is one of the common malignancies. There is sufficient evidence that sunlight (ultraviolet radiation) contributes to the development of skin cancer, but there is also evidence that relates adequate serum levels of vitamin D produced on the skin by the action of ultraviolet radiation with the decreased risk of various types of cancers, including skin cancer. The aim of this study was to investigate the association of vitamin D serum levels among patients with non-melanoma skin cancers (basal cell carcinoma and squamous cell carcinoma) and controls.

**Methods:**

A prospective observational case-control study was conducted in a sample of 84 subjects in Extremadura (Spain). Forty-one patients with histologically diagnosed basal cell carcinomas and squamous cell carcinomas and 43 healthy controls were randomly chosen to assess whether vitamin D (25(OH)D_3_) serum level, age and sex were related to non-melanoma skin cancer and to determine the possible risk of this type of skin cancer for these variables.

**Results:**

When analysing serum vitamin D levels, we ensured that all our subjects, both cases and controls, had normal or low serum vitamin D levels, even though the samples were taken during months with the highest solar irradiance in our region. It is striking in our results that there was a higher percentage of subjects with deficits of vitamin D who did not have skin cancer (66%) than patients with deficits with these types of skin cancers (34%). When adjusting the model for age and sex, vitamin D values above 18 ng/ml increased the risk of suffering from non-melanoma skin cancer by nearly 7-fold (aOR: 6.94, 95% CI [1.55–31.11], *p* = 0.01).

**Conclusions:**

Despite the controversial data obtained in the literature, our results suggest that lower levels of vitamin D may be related to a reduced incidence of non-melanoma skin cancer.

## Introduction

Non-melanoma skin cancer (NMSC) refers to all types of cancer that occur in the skin and are not melanoma. Several types of skin cancer fall within the broader category of NMSC, with the most common types being basal cell carcinoma (BCC) and squamous cell carcinoma (SCC) (Griffin, Ali & Lear, 2016; Lomas, Leonardi-Bee & Bath-Hextall, 2012).

Non-melanoma skin cancers are considered the most frequent cancers (American-Cancer-Society, 2021; Griffin, Ali & Lear, 2016; Siegel et al., 2021), but many countries do not record the incidence of BCC or SCC, recording them only as a part of NMSC. Unfortunately, this category can include other skin tumours. Moreover, of those that do register and record incidence of non-melanoma skin cancers, some rely on notification, with or without histological proof. BCC and SCC commonly occur as multiple tumours in a single person. Studies may count the number of people with lesions or the number of lesions, so care must be taken when using these data. In addition, most studies are performed in predominantly white populations, so the incidence and risk factors for NMSC in black populations are even less clear. However, the incidence is rising by 3–7% per year in most countries (Lomas, Leonardi-Bee & Bath-Hextall, 2012; Lucas et al., 2006). The primary difficulty in measuring the incidence arises from poor registration practices in the majority of countries, as happens in Spain (Tejera-Vaquerizo et al., 2016).

The etiopathogenesis of these skin cancers seems to be multifactorial: both intrinsic and environmental factors are involved. There is evidence supporting the hypothesis that ultraviolet radiation causes skin cancer (Narayanan, Saladi & Fox, 2010), but at the same time, there is sufficient scientific evidence indicating the protective effect of vitamin D produced in the skin by ultraviolet radiation against different types of cancers, including NMSC (Reichrath, Saternus & Vogt, 2017). This paradoxical effect can be explained by evidence suggesting that the skin’s synthesis of vitamin D is self-limited and fades after five to ten minutes, while longer durations of sun exposure elevate the risk of skin cancer (IARC, 2008; Reichrath, 2006).

BCC and SCC are directly correlated with the accumulation of sun exposure on the skin over years. In contrast, cases of melanoma (and some BCCs) are slightly different. The pattern believed to induce melanoma is one of brief, intense sun exposure rather than damage from years of tanning (Ananthaswamy, 2001).

Apart from the classical actions in mineral metabolism, vitamin D performs other non-classical functions, such as the regulation of cell proliferation and differentiation in many cell types. Specifically, there are numerous functions of the skin regulated by vitamin D and/or its receptor (vitamin D receptor: VDR). Among them, we found inhibition of proliferation (Cordero et al., 2002), promotion of immunosuppression (Dusso, Brown & Slatopolsky, 2005), regulation of the follicular cycle (Bikle et al., 2006; Hochberg et al., 1985; Marx, Bliziotes & Nanes, 1986), suppression of tumour formation (Bikle, 2011) and stimulation of differentiation (Bikle, 2012; Bikle, Oda & Xie, 2004).

Laboratory studies suggest that vitamin D and its metabolites may reduce the risk of skin cancer. However, only a few epidemiological studies have assessed the association between vitamin D serum levels and NMSC risk, and these have shown controversial results: some report an association between higher vitamin D levels and an increased risk of developing skin cancer (Asgari et al., 2010; Eide et al., 2011; Liang et al., 2012; Soares et al., 2018; van der Pols et al., 2013; Vojdeman et al., 2019), while others report a decreased risk (Tang et al., 2011; Tang et al., 2010), and others found no association (van Dam et al., 2000; van Deventer, Kannenberg & du Toit, 2018). In 2014, Caini et al. (2014) found a statistically significant positive association with an increased risk of NMSC for high values of 25(OH)D_3_ in their review and meta-analysis. Their findings seem to suggest that vitamin D intake *via* foods and/or supplements is neither associated with elevated skin cancer risk nor exerts any protective effect against skin cancer development; however, it is unclear whether an amount of UVB radiation exists that could ensure a health benefit without increasing the risk of skin cancer, suggesting that further research is needed.

Serum levels of 25(OH)D_3_, and not 1,25(OH)_2_D_3_, are the best indicator of vitamin D status. Optimal serum levels of 25(OH)D_3_ range between 30 and 60 ng/ml. Levels between 20 and 30 ng/ml are considered low, and levels between 60 and 100 ng/ml are considered high but still within the normal range. Values less than 20 ng/ml indicate a deficit of vitamin D, while values between 100 and 150 ng/ml are considered an excess of vitamin D (Holick, 2007).

The National Institutes of Health (NIH) in the United States (US) concludes that vitamin D levels equal to or greater than 30 ng/ml are adequate for cancer prevention (Bischoff-Ferrari et al., 2006; Gorham et al., 2005). However, according to clinical practice guidelines of the American Society for Endocrinology (Endocrine Society Practice Guidelines), serum vitamin D levels should not be investigated in patients without risk factors, as there is no scientific evidence for the benefit of screening for vitamin D in the general population (Tang et al., 2012). Efforts are currently being made to determine the optimal 25(OH)D_3_ serum levels (and the intake of vitamin D needed to obtain them) for the prevention of cancer, as the pharmacological modulation of the endocrine system of vitamin D represents a promising strategy for cancer prevention and treatment and more specifically for skin cancer (Neale et al., 2016; Waterhouse et al., 2019). In this regard, the International Agency for Research on Cancer (IARC), which is part of the World Health Organization (WHO), considers that setting a lower limit of 20 or 30 ng/ml is currently inappropriate, as there are no randomized studies suggesting that maintaining those levels of 25(OH)D_3_ can prevent any type of cancer or any other pathology. For this reason, they consider it premature to change the recommendations on the intake of vitamin D, added to the fact that there is insufficient evidence regarding the lack of harm due to prolonged intake vitamin D (IARC, 2008).

Apart from understanding the genetic basis of skin cancer, being able to modify environmental factors, such as vitamin D intake or sun exposure, is extremely important for the prevention and prognosis of this pathology. This is why we designed a study in which we assessed the relationship between NMSC and serum vitamin D levels. The objective of our study was to evaluate whether vitamin D levels have a differential effect in patients with NMSC *versus* healthy subjects and to determine the possible risk of NMSC for the variables sex, age and vitamin D levels and their model fit. Our study aims to provide knowledge so that in future research, it could be possible to propose new measures regarding optimal levels of vitamin D that could help prevent or improve the prognosis of NMSC.

## Materials & Methods

### Study design and ethics

This was a prospective observational case-control study performed in Extremadura (a region located in southwestern Spain) at the Plastic Surgery Service of the Cáceres University Hospital Complex (the only reference Centre in Extremadura for Plastic Surgery).

Each subject provided written informed consent before entering the study, which met the requirements of the World Medical Association and the Declaration of Helsinki. This study was approved by the local Ethics Committee and the Institutional Review Board (IRB) of the University of Extremadura (protocol ID number: 74/2015 - approved on May 26th, 2015). The correspondence between codes and subjects’ identification data was kept in a separate location that was only accessible to the coordinating investigator and/or investigators responsible for the centre or to the corresponding authorities of the hospital Clinical Research Ethics Committees (CREC, approved on April 29th, 2015). This study was reported following the Strengthening the Reporting of Observational Studies in Epidemiology (STROBE) guidelines for reporting observational studies (von Elm et al., 2007).

### Study procedures

We performed intentional non-probabilistic sampling. The recruited subjects were those who attended our outpatient plastic surgery consultation. The cases included patients suspected of suffering from NMSC. Apart from skin cancer, these subjects were otherwise healthy. They were recruited when surgery was proposed, after the clinical diagnosis. The suspected skin cancers were excised 2 weeks later, and at the same time peripheral venous blood samples were drawn. We also enrolled the control subjects in our outpatient plastic surgery consultation. They were healthy patients that consulted for other medical consultations, such as traumatic wounds or burns, and peripheral venous blood samples were drawn during this first visit after being thoroughly examined - photoexposed and non-photoexposed areas - by an experienced plastic surgeon to confirm that they did not have any type of skin cancer. Data were collected as previously described in other study from our research group (Morgado-Aguila et al., 2020). Subsequently, of all the recruited patients, the selection was randomized using the SPSS program to generate random numbers (SPSS, Chicago, IL, USA).

Both cases and controls had similar sun exposure habits, and they all come from Extremadura, a mostly rural region in Spain with high solar radiation (latitude 40°).

Only patients over 18 years of age with BCC or SCC were included. Exclusion criteria for cases and controls included history of treatment with immunosuppressive drugs, having received an organ transplant or taking vitamin supplements in any form. We also excluded subjects with non-malignant lesions or other types of skin cancer or if we identified the coexistence of BCC or SCC.

Calculations of minimally detectable effect sizes, as well as of sample sizes for the present study, were performed using G*Power software (version 3.1.9, Universität Düsseldorf, Düsseldorf, Germany) (Faul et al., 2009). For our sample size (controls, *n* = 43, cases, *n* = 41), we calculated the statistical power to detect medium effect sizes (anticipated Cohen’s *d* = 0.60/OR = 3) *α* = 0.05, obtaining an output of 0.88.

We chose to divide the age variable in ≤70 years old and >70 years old (≤70 age/>70 age) according to the closest cut-off value in relation to the mean (66.90) and median (66.50) of that variable in our study sample.

### Study population and sample size

We initially evaluated 93 subjects. Cases and controls were recruited during 2016 and 2017 in the Plastic Surgery Department in Cáceres (Spain). The patient group consisted of 50 randomly selected Caucasian subjects with suspected non-melanoma skin cancer, and additionally 43 healthy Caucasian individuals were enrolled to serve as controls.

To avoid selective pressure (selection bias) in our sample, given that sex and age are variables related to the occurrence of the disease and that these variables were intended to be studied, we adopted the criterion of random selection of subjects with the same inclusion and exclusion criteria in both groups. A total of 33 subjects were males (controls, *n* = 9, cases, *n* = 24), and 51 were females (controls, *n* = 34, cases, *n* = 17).

**Figure 1:**
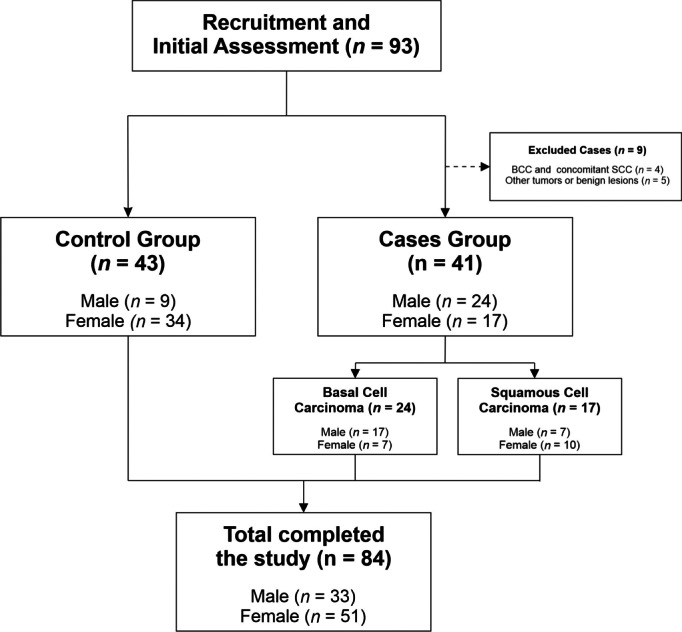
CONSORT flowchart of recruited patients. The figure describes the study’s participant selection process, showing the number of participants at each stage.

Twenty-four patients had BCC and 17 had SCC. A total of 9 subjects were excluded, 5 of them because they had a non-malignant lesion or other type of skin cancer, and 4 of them because they had or previously had both skin cancers (BCC and SCC). Figure 1 illustrates the participant selection process.

### Skin samples

Surgical excision was always performed by the same surgeon according to previous protocols (Brodland & Zitelli, 1992; Gulleth et al., 2010), and the skin samples were sent to the Pathology Laboratory for Histologic Study in a tertiary-level hospital. Pathologists studied the samples as part of their routine work, and they did not know that the patients were being studied for any reason other than clinical management.

### Vitamin D measurement

**Figure 2:**
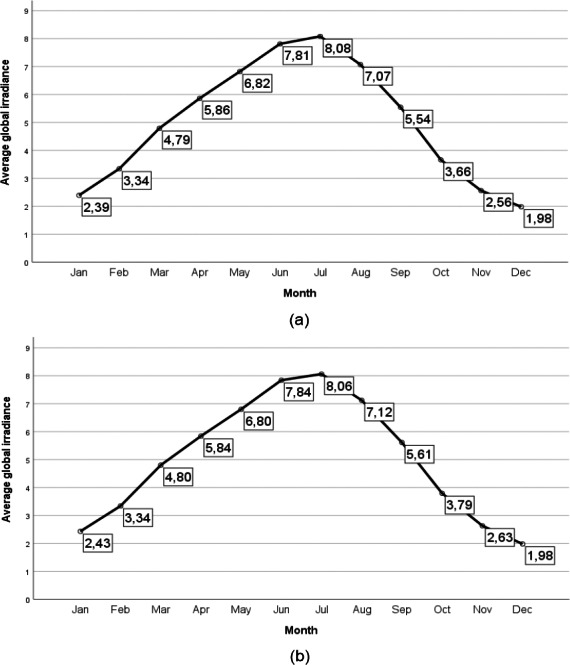
Average global irradiance in the provinces of Cáceres and Badajoz (Spain). Average global irradiance in the province of Cáceres (Spain) (A) and average global irradiance in the province of Badajoz (Spain) (B). The radiative flux is expressed in KWh *m*^−2^ día ^−1^. The source of data a was obtained from the State Meteorological Agency of Spain (SanchoÁvila et al., 2012).

Patients and controls were randomly chosen. However, to avoid biases regarding the measurement of vitamin D due to seasonal variations in serum vitamin D levels and possible differences in terms of solar radiation, all samples were recruited between May and August (2016 and 2017), that is, during the four months of the year with the highest solar radiation in our region (Fig. 2).

Blood specimens in both cases and controls were drawn in red stopper tubes, which contained no anticoagulant or preservative, and were subsequently frozen at −90 °C.

An independent laboratory (STAB—Servicio de Técnicas Aplicadas a la Biociencia) in the nearby city of Badajoz (Spain) assessed the vitamin D levels in the peripheral venous blood samples. The aim of our study and the sample identification and categorization (case or control) were unknown to the laboratory personnel.

To determine plasma levels of vitamin D (25(OH)D_3_), they used the Human 25(OH) Vitamin D ELISA kit from Abbexa Ltd., and 50 µl of undiluted sample were used for each point.

First, we stratified the absolute plasma levels of vitamin D provided by the laboratory into groups according to values commonly used (Holick, 2007): <20 ng/ml (deficit), 20–30 ng/ml (low limit), 30–60 ng/ml (normal), 60–100 ng/ml (high limit), 100–150 ng/ml (excess), and >150 ng/ml (intoxication). However, in our sample, the upper levels of the variable were not reached. For this reason, the vitamin D variable was restratified post hoc into two categories: ≤ 18 ng/ml and >18 ng/ml. We chose to divide the vitamin D variable according to the nearest rounded cut-off value in relation to the mean (19.28) and median (18.08) of that variable in our study sample, which was 18 ng/ml (≤18 ng/ml/>18 ng/ml).

### Study outcomes

The aim of this study was to assess whether vitamin D levels have a differential effect in patients with NMSC *versus* healthy subjects and to evaluate their possible protective or risk effects. The primary outcome of the study was to assess differences in vitamin D levels between the study groups (cases *vs.* controls). Secondary outcomes were to determine the possible risk of NMSC for the variables sex, age and vitamin D levels and their model fit.

### Statistical methods

Initially, a descriptive analysis of serum vitamin D levels was performed in relation to the variables gender, age, and tumour type. The normality of the data was tested using the Shapiro–Wilk test, and vitamin D and age were not normally distributed.

Likewise, the *χ*^2^ test was used to evaluate the relationship between the type of tumour and vitamin D levels.

Binary logistic regression was employed to calculate the odds ratios (OR) and 95% confidence intervals (95% CI) to assess the risk of skin cancer for vitamin D serum level, age, and sex among the subjects.

For other analyses, Fisher’s test was used for categorical data, Student’s *t*-test was used for continuous parametric data, and the Mann–Whitney and Kruskal-Wallis tests were used for continuous non-parametric data. The significance threshold was set at two-sided alpha = 0.05 for all analyses.

All statistical analyses were performed using SPSS for Windows, version 24.0 (IBM Corporation, Armonk, NY, USA).

## Results

### Study sample

A total of 84 subjects were included in our study. The patient group consisted of 41 subjects with NMSC, and 43 healthy individuals were enrolled to serve as controls. A total of 33 (39.3%) were males, and 51 (60.7%) were females.

Our subjects were between 38 and 93 years old with a mean age of 66.9 ± 14.81 years (males: 70.55 ± 13.67; females: 64.55 ± 15.16). The mean age of the control subjects was 56.79 ±  10.78 years and that of the BCC and SCC patients was 76.50 ±  8.57 and 78.94 ± 12.63 years, respectively.

### Vitamin D levels in cases and controls

Table 1 shows baseline serum vitamin D levels of the study groups. A total of 59.5% of our sample had serum vitamin D levels below 20 ng/ml, placing them in deficit according to the prescribed standards; 22 patients (26.2%) had serum vitamin D levels in the range of 20-30 ng/ml (low limit), and 12 patients (14.3%) had levels between 30–60 ng/ml (normal). Levels above stratum 30–60 ng/ml were not observed in our sample. A total of 67.4% of the subjects in the control group had serum vitamin D levels below 18 ng/ml, while 70.7% of the cases (BCC/SCC) had levels above 18 ng/ml.

**Table 1:** Baseline characteristics of the sample groups.

	**Total sample (%)**	**Controls (%)**	**Cases (%)**	**BCC**	**SCC**
				**Cases (%)**	**Cases (%)**
**Vitamin D** ^a^					
<20 ng/ml (Deficit)	50 (59.5)	33 (76.7)	17 (41.5)	10 (41.7)	7 (41.2)
20-30 ng/ml (Low Limit)	22 (26.2)	7 (16.3)	15 (36.6)	9 (37.5)	6 (35.3)
30-60 ng/ml (Normal)	12 (14.3)	3 (7)	9 (22)	5 (20.8)	4 (23.5)
**Vitamin D** ^b^					
≤18 ng/ml	41 (48.8)	29 (67.4)	12 (29.3)	8 (33.3)	4 (23.5)
>18 ng/ml	43 (51.2)	14 (32.6)	29 (70.7)	16 (66.7)	13 (76.5)
**Sex**					
Male	33 (39.3)	9 (20.9)	24 (58.5)	17 (70.8)	7 (41.20)
Female	51 (60.7)	34 (79.1)	17 (41.5)	7 (29.2)	10 (58.8)
**Age**					
≤70 age	47 (56)	39 (90.7)	8 (19.5)	5 (20.8)	3 (17.6)
>70 age	37 (44)	4 (9.3)	33 (80.5)	19 (79.2)	14 (82.4)

**Notes.**

Data are expressed as frequencies (percentages) within the control and case groups.

Abbreviations BCC Basal Cell Carcinoma SCC Squamous Cell Carcinoma

a Standard vitamin D stratification (Holick 2007), values above stratum 30–60 ng/dl were not found in our sample.

b Stratification carried out according to the cut-off values defined in our sample.

Serum vitamin D levels for each group (controls, BCC cases and SCC cases) are shown in Table 2. The mean and standard error of the mean (SEM) vitamin D levels in the study sample were 19.28 ± 1.03 ng/ml (range 43.4). For sex and age, there were no statistically significant intra-group differences (Controls and Cases) in vitamin D levels. The highest serum vitamin D levels were observed in the case group. No statistically significant differences in serum vitamin D levels were observed in the case group (BCC/SCC). The mean and SEM of serum vitamin D levels in the case and control groups were 23.48 ± 1.31 ng/ml and 15.28 ± 1.34 ng/ml, respectively.

**Table 2:** Vitamin D levels in sample groups.

	**Controls**			**BCC**			**SCC**		
				**Cases**			**Cases**		
	**Mean**	**SEM**	***p*** **-Value**	**Mean**	**SEM**	***p*** **-Value**	**Mean**	**SEM**	***p*** **-Value**
**Sex**									
Male	15.82	3.12	0.92	22.25	2.08	0.49	26.16	3.33	0.36
Female	15.14	1.50		24.91	2.88		22.68	2.80	
**Age**									
≤70 age	15.53	1.43	0.51	22.15	4.73	0.95	24.35	5.81	0.77
>70 age	12.93	3.58		23.26	1.80		24.06	2.36	

**Notes.**

25(OH)D_3_ mean levels are expressed in ng/ml. *p*-Values were performed using the Mann–Whitney *U*-test.

Abbreviations BCC Basal Cell Carcinoma SCC Squamous Cell Carcinoma SEM Standard Error of the Mean

**Figure 3:**
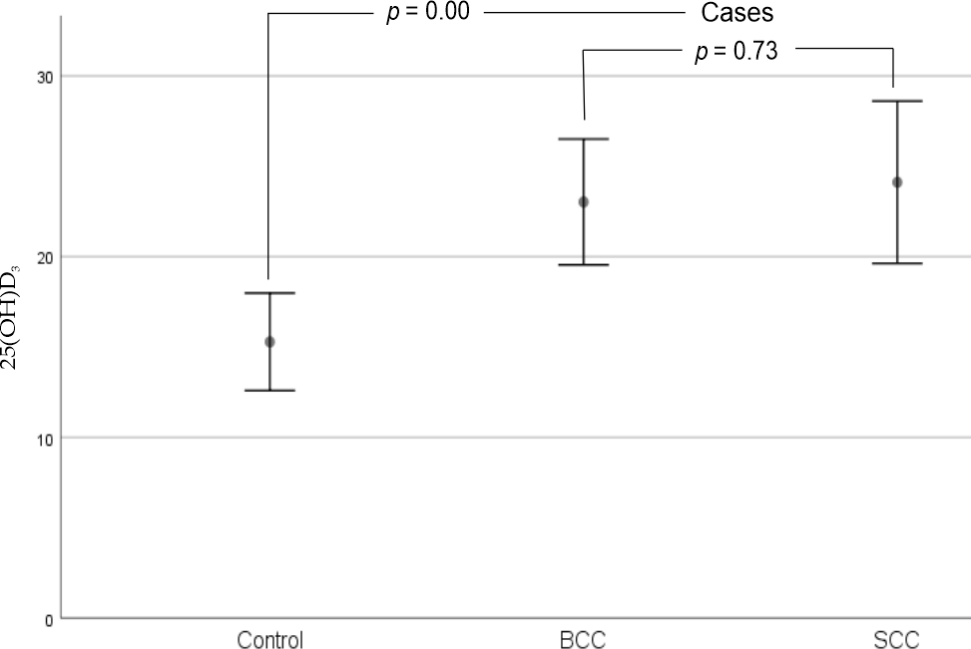
Error bars for mean 25(OH)D_3_ levels of cases and controls (95% CI). There were statistically significant differences when comparing BCC and SCC cases to controls with respect to serum vitamin D levels in our sample. No statistically significant differences in serum vitamin D levels were observed between cases (BCC and SCC). Abbreviations: BCC, Basal Cell Carcinoma; SCC, Squamous Cell Carcinoma. *p*-Values were performed using the Mann–Whitney *U*-test.

However, as shown in Fig. 3, we observed that there were statistically significant differences when comparing BCC (23.02 ± 1.68 ng/ml) and SCC (24.11 ±2.12 ng/ml) cases to controls (15.28 ± 1.34 ng/ml) with respect to serum vitamin D levels in our sample (*p* = 0.00).

### Vitamin D, age and sex and non-melanoma skin cancer risk

We used the binary logistic regression test to assess whether vitamin D serum level, age or sex were related to skin cancer risk. The vitamin D variable was dichotomized (according to the cut-off values defined in our sample). Serum vitamin D levels above 18 ng/ml increased the risk of suffering from NMSC up to 5-fold (OR: 5.01, 95% CI [1.99–12.65], *p* = 0.00) compared to levels below 18 ng/ml. Moreover, when adjusting for vitamin D levels, sex and age, vitamin D accounted for nearly a 7-fold increase in the risk for developing NMSC (aOR: 6.94, 95% CI [1.55–31.11], *p* = 0.01) (Table 3).

**Table 3:** Association between vitamin D level, sex, age, and non-melanoma skin cancer.

	**OR (95% CI)**	***p*** **-Value**	**aOR (95% CI)**	***p*** **-Value**
Vitamin D (>18 ng/ml reference)	5.01 (1.99–12.65)	0.00	6.94 (1.55–31.11)	0.01
Sex (Female reference)	0.19 (0.07–0.49)	0.00	0.11 (0.02–0.54)	0.00
Age (>70 age reference)	40.22 (11.19–145.62)	0.00	58.59 (11.26–304.90)	0.00

**Notes.**

Data shown as ORs (95% CIs) and *p*-values were obtained from binary logistic regression.

Abbreviations 95% CI 95% confidence intervals OR odds ratio aOR odds ratio adjusted for vitamin D levels, sex, and age; the reference category is the “control group”

It was also observed that being over 70 years of age increased the risk of developing NMSC, with this variable being the one that predicted the strongest association (OR: 40.22, 95% CI [11.19–145.62], *p* = 0.00). The sex variable was associated with a protective factor for females over males in the occurrence of NMSC (OR: 0.19, 95% CI [0.07–0.49], *p* = 0.00). In our sample, when adjusting for vitamin D levels, sex and age, data implied that the female sex was protected against NMSC (aOR 0.11, 95% CI [0.02–0.54], *p* = 0.00), and being over 70 years of age increased the risk of NMSC by up to 58-fold (aOR 58.59, 95% CI [11.26–304.90], *p* = 0.00) (Table 3).

Omnibus tests of coefficients of the binary logistic regression model were significant for the variables vitamin D serum level, sex, and age. The model obtained a good fit of the variables, with a Nagelkerke *R*-squared of 0.72. The OR values increased in the adjusted model (aOR), as shown in Table 3.

## Discussion

It is well known that sex and age are related to NMSC (Garcovich et al., 2017; Joseph, Mark & Mueller, 2001; Oberyszyn, 2008; Pascual et al., 2004; Scrivener, Grosshans & Cribier, 2002). Consistently with previous studies, our study parallelly illustrates the fact that female sex serves as a protective factor NMSC, conversely, age above 70 augments the risk of NMSC.

Vitamin D levels in our sample were normal or low in any case, regardless of seasonal influence. This may be part of the so-called vitamin D deficiency pandemic. Vitamin D deficiency and insufficiency are global health issues that need to be addressed, such as a pandemic, since they afflict more than one billion children and adults worldwide. They have become a major public health interest due to their association with acute and chronic illnesses, including cardiovascular disease, infectious diseases, diabetes mellitus, neurological disorders, osteoporosis, fractures, autoimmune diseases, and cancer. Improving vitamin D status worldwide would have remarkable effects on public health (Cashman et al., 2016; Holick, 2010; Holick, 2017; Holick & Chen, 2008).

There is controversy as to the optimal serum vitamin D levels that could be protective against cancer (Bischoff-Ferrari et al., 2006; Gorham et al., 2005; IARC, 2008). Low vitamin D serum levels were related to a lower incidence of NMSC in our sample. Vitamin D serum levels greater than 18 ng/ml may increase the risk of NMSC.

We want to emphasize that, as derived from the studies performed for skin cancer or even for other types of tumours, such as breast, colorectal or prostate cancer, vitamin D serum levels cannot be considered an independent risk factor for NMSC. It seems probable that other factors, such as ethnicity, phenotype, 25(OH)D_3_ plasma levels, VDR gene polymorphisms and UV radiation exposure, are confounding factors that introduce heterogeneity (Giovannucci, 2005; Gnagnarella et al., 2020; Mahamat-Saleh, Aune & Schlesinger, 2020).

The best indicator of vitamin D status is 25(OH)D_3_ serum level. If it is not possible to assess this variable in a vacuum due to the factors that could influence 25(OH)D_3_ status, such as diet or dietary supplements, sun exposure (UV radiation) or body mass index (Wortsman et al., 2000), which should also be assessed. Even though the measurement thus far accepted is the measurement of 25(OH)D_3_, news online (no scientific publications thus far) includes medical personalities stating that determining only vitamin D_3_ values can lead physicians to make incorrect decisions. This could happen in the case of people who are vegetarians, for example, who tend to have low levels of D_3_ and yet high vitamin D_2_ levels. The same can happen in people who suffer some type of pathology as in patients with biliary cirrhosis, intestinal problems or in cases of celiac disease (Organización Médical Colegial, 2009). In our study, we considered the best variable in terms of biases to be the plasma levels of 25(OH)D_3_ for trying to assess the relationship between vitamin D plasma levels and the risk of BCC or SCC in our population.

As stated in the previous sections that there are few published works linking vitamin D levels and NMSC, with inconsistent results (Asgari et al., 2010; Caini et al., 2014; Eide et al., 2011; Liang et al., 2012; Soares et al., 2018; Tang et al., 2011; Tang et al., 2010; van Dam et al., 2000; van der Pols et al., 2013; van Deventer, Kannenberg & du Toit, 2018; Vojdeman et al., 2019). We consider this to be a limitation for our study since we cannot design it from a more generalized hypothesis. In this regard, before accomplishing our work, we expected to find, based on information obtained from the bibliography collected and that had been exposed thus far in this text, studies that supported the protection of vitamin D against skin cancer.

In our bibliographic search, we found very few articles focusing on the possible relationship between vitamin D serum levels and NMSC risk and with very contradictory results.

For example, in a case-control study performed in older American men, a lower incidence of NMSC was found in subjects with higher concentrations of pre-vitamin D compared to subjects with lower concentrations (Tang et al., 2010). Lesiak et al. (2011) also found that mean serum 25(OH)D_3_ was significantly higher in a control group of Polish origin than in cases of BCC.

Nevertheless, similar to the results obtained in our study, we also found opposing observations to this previous study. For example, a study in a Brazilian sample has been recently published in which it is concluded that patients with NMSC presented higher 25(OH)D_3_ serum levels than controls, although even with those higher levels, they theoretically still had deficits of vitamin D (Soares et al., 2018). A similar finding was published by Eide et al. in a study of a sociodemographically diverse population in Detroit (Eide et al., 2011), who observed a relationship between higher (but not increased) 25(OH)D_3_ serum levels and the risk of developing NMSC. Vojdeman et al. (2019) conducted a study on the Danish population and found higher levels of vitamin D in relation to an increased incidence of melanoma and NMSC.

In 2009, the authors of one of the multiple meta-analyses on vitamin D and skin cancer performed a bibliographic search looking for publications that studied the relationship between melanoma, NMSC, VDR gene polymorphisms, intake of vitamin D and serum levels of 25(OH)D_3_ (Gandini et al., 2009). Specifically, in this meta-analysis, they found interesting results regarding polymorphisms and NMSC. In particular, VDR gene *FokI* and *BsmI* polymorphisms were associated with NMSC, even if in different directions. The *FokI f* allele exhibited a significant positive association with NMSC, while the *BsmI B* allele displayed a significant negative association with NMSC. However, they concluded that the association of vitamin D intake was less clear and that more studies were needed to clarify the role of diet in skin cancer.

Initially, we hypothesized that vitamin D serum levels would be normal or elevated, not deficient, in our patients due to the seasonal influence of sampling. However, despite living in a country with high solar radiation (40° latitude), all subjects had normal or low vitamin D levels, and no subject had elevated levels. The highest percentage of subjects (59.5%) was conspicuously deficient in vitamin D, despite having been studied in the four months with the highest solar radiation in our region of Extremadura (Fig. 2). This may be due to the so-called vitamin D deficiency pandemic (Cashman et al., 2016; Holick, 2010; Holick & Chen, 2008).

Indeed, we did not expect to observe more patients with deficiency of vitamin D in the three subgroups, but it is especially striking that, considering the supposed protective role of vitamin D in skin cancer, we found a greater number of patients with deficit that did not present tumours than even the sum of patients in deficit with either of the two types of tumours (Table 1). In fact, there was a significant difference between the levels of vitamin D and whether the subject suffered from skin cancer: the lowest levels of vitamin D were related to a lower incidence of NMSC in our sample.

Currently, based on IARC recommendations (IARC, 2008), physicians do not prescribe NMSC patients vitamin D supplements. In fact, according to the Endocrine Society Practice Guidelines, serum vitamin D levels should not be investigated in patients without risk factors, as there is no scientific evidence for the benefit of vitamin D screening in the general population (Tang et al., 2012). To change recommendations on vitamin D levels across heterogeneous populations in terms of phototype, latitudes, intake of vitamin D from the diet and habits of sun exposure, clinical trials are needed, so that these recommendations will be truly evidence-based (Caini et al., 2014).

Based on our bibliographic search and results, we believe it is necessary to develop more epidemiological and experimental studies to elucidate the intricate relationship between vitamin D levels and the development of cancer, specifically skin cancer, in terms of sunscreen use, dietary changes, or possible use of vitamin D supplements or their analogues. Despite the striking results obtained in our work, we cannot propose any other new preventive or therapeutic measures regarding non-melanoma skin cancers in relation to vitamin D optimal serum levels.

We are aware that our study is not without limitations: we could only collect a limited number of participants, we were unable to match the ages in cases and controls, solar irradiances were measured indirectly using data from the Spanish Meteorological Agency (AEMET), and we did not control for individual irradiances or personal or professional factors that could alter sun exposure, and therefore vitamin D levels, of individuals.

However, we believe it is necessary to continue the current line of investigation, since it could lead to a radical change in the prevention and prognosis of this type of cancer.

## Conclusions

The present study demonstrates that low vitamin D levels are associated with a lower incidence of BCC and SCC. Vitamin D serum levels above 18 ng/ml may increase the risk of NMSC.

Our results provide useful information for acquiring further knowledge on the relationship between vitamin D and NMSC. Further studies with a larger number of participants and more information about possible confounding factors are needed to confirm or reject our results.

## Supplemental Information

10.7717/peerj.12234/supp-1 Supplemental Information 1 Raw Data

## Additional Information and Declarations

### Data Availability

The following information was supplied regarding data availability: The raw measurements are available in the Supplementary File.
